# Rapid Micromolding of Sub-100 µm Microfluidic Channels Using an 8K Stereolithographic Resin 3D Printer

**DOI:** 10.3390/mi14081519

**Published:** 2023-07-28

**Authors:** Arpith Vedhanayagam, Michael Golfetto, Jeffrey L. Ram, Amar S. Basu

**Affiliations:** 1 Electrical and Computer Engineering, Wayne State University, Detroit, MI 48202, USA; 2 Department of Physiology, Wayne State University School of Medicine, Detroit, MI 48201, USA

**Keywords:** 3D resin printing, micromolding, rapid prototyping, PDMS soft lithography

## Abstract

Engineering microfluidic devices relies on the ability to manufacture sub-100 micrometer fluidic channels. Conventional lithographic methods provide high resolution but require costly exposure tools and outsourcing of masks, which extends the turnaround time to several days. The desire to accelerate design/test cycles has motivated the rapid prototyping of microfluidic channels; however, many of these methods (e.g., laser cutters, craft cutters, fused deposition modeling) have feature sizes of several hundred microns or more. In this paper, we describe a 1-day process for fabricating sub-100 µm channels, leveraging a low-cost (USD 600) 8K digital light projection (DLP) 3D resin printer. The soft lithography process includes mold printing, post-treatment, and casting polydimethylsiloxane (PDMS) elastomer. The process can produce microchannels with 44 µm lateral resolution and 25 µm height, posts as small as 400 µm, aspect ratio up to 7, structures with varying z-height, integrated reservoirs for fluidic connections, and a built-in tray for casting. We discuss strategies to obtain reliable structures, prevent mold warpage, facilitate curing and removal of PDMS during molding, and recycle the solvents used in the process. To our knowledge, this is the first low-cost 3D printer that prints extruded structures that can mold sub-100 µm channels, providing a balance between resolution, turnaround time, and cost (~USD 5 for a 2 × 5 × 0.5 cm^3^ chip) that will be attractive for many microfluidics labs.

## 1. Introduction

Over the last three decades, the field of microfluidics has become a transformative technology for diverse applications, including point-of-care diagnostics, analytical chemistry, cell and biomolecular assays, and organ on a chip. A key enabling capability is the ability to fabricate channels and circuits ranging from 10 to 100 microns in size [1,2]. Historically, microfluidic chips have often required microfabrication facilities, capital investment in equipment, and expertise in photolithographic processing typically employed in microelectronic circuit fabrication. Furthermore, many assays place specific material constraints such as optical transparency, chemical inertness, high thermal resistance, biological compatibility, and relatively low non-specific adsorption. 

Early generations of microfluidic chips were composed of glass [1], which continues to be used for scaled production [3]. Glass is optically transparent, chemically inert, and has high biological compatibility. Recent generations of commercial chips are often made of polycarbonate, polymethylmethacrylate (PMMA), and other polymers fabricated using high-resolution injection molding [4]. Both methods are time-consuming and require capital equipment and consumables for each chip, making rapid prototyping a challenge [3,5]. 

Since the late 1990s, rapid prototyping of microfluidic chips has relied heavily on polydimethylsiloxane (PDMS), a transparent elastomer offering lower cost and time for prototyping compared to glass [6]. The widely used soft lithography fabrication process involves casting the liquid pre-polymer over a high-fidelity mold composed of SU8 or other photoresists [7]. The process begins with spin coating a layer of SU8 on a polished silicon wafer, aligning a mask on the SU8, exposure to UV light, followed by development. For multilayer molds, this process is repeated for each layer, and registration of layers is required to align structures. Once the PDMS has been cast over the silanized mold, it is allowed to cure, forming the negative image of the mold. The PDMS is bonded to a second layer of PDMS or glass to form a sealed channel. 

While the soft lithography process can create channels as small as 50 nm [8], one limitation is that the channel height is essentially set by the thickness of the SU8 layer, making it difficult to fabricate channels with multiple heights or smooth transitions in height. A second limitation is that mold fabrication typically requires clean room facilities with a mask aligner and a spin coater. Third, many labs do not have access to a mask maker or high DPI printer; hence, the photolithography mask must be ordered from a vendor, with a typical turnaround time of 3–5 days. Although the lithography process itself takes only a few hours, the practical turnaround time is often one week or more. 

Rapid prototyping methods have been developed to reduce design iteration times and accelerate productivity in device engineering. We refer the reader to the detailed review in [9] and highlight a few here. For example, a Cricut craft cutter can carve openings in thin films such as double-sided tape, which can be stacked with other layers such as glass and PDMS [10] to form microfluidic channels. This method is both fast and economical, but the minimum channel width is 200 µm [10]. CO_2_ lasers can ablate channels on materials such as PMMA, PET film, silicone, and mylar films [11] and have been used to make microfluidic devices such as mixers for Ammonia detection [12]. This method is also fast; however, the minimum channel width is 250 µm [12], and the typical cost of CO_2_ laser cutters is >USD 2000. Inkjet printing of paper microfluidics has been employed to rapidly prototype microfluidic devices such as immunoassays, cell traps, and pollutant detection devices [13]. The minimum channel width is 150 µm [13]. With the Cricut plotter, laser cutter, and inkjet printing, we can vary the z-axis shape, but this would require specialized equipment to align the different layers.

In recent years, 3D printing has become both accessible and cost-effective, while performance and resolution have steadily improved [14]. The strengths of 3D printing are that it does not require clean rooms or masks, and it enables capabilities such as varying channel height and fabricating integrated reservoirs and fluidic interconnects.

The most popular form of additive 3D printing is fused deposition modeling (FDM), an additive method where thermoplastic materials, heated above their glass transition temperature, flow through a nozzle controlled by an XYZ stage [14]. The minimum structure is determined by the size of the nozzle and material properties. For example, 254 µm lines can be printed using the Stratasys Dimension 768 printer with a 0.01 nozzle [15]. A 0.25 mm nozzle can make structures as small as 40 µm [16]. FDM has been used to make capillary valves for centrifugal microfluidics, where the valves are printed in ABS and fused to PMMA [15]. FDM can print a wide range of thermoset plastics. However, due to the nature of extrusion-based printing, the surface roughness is compromised, dimensional accuracy is limited by the XYZ stage, and the method cannot generate the rectangular channel profiles often desired in microfluidics. Due to surface roughness, PDMS casts made from FDM molds have poor optical qualities.

In inkjet 3D printing (i3DP), also known as binder jetting, a layer of powdered particles is laid on a print bed with a leveling roller. A print head deposits droplets of resin, which are used to fuse the powdered particles [14,17]. This method is used to print molds from which PDMS can be cast. Structures that are 250 × 250 µm can be printed reliably using this technology [18] and have been proven to print microfluidic mixers that are multilayered and have varying patterns in different parts of the channel [16]. The print dimensions are dependent on the size of the droplet and the spacing of the nozzles [19]. The molds printed have rough edges due to printing resolutions, compromising optical quality similar to FDM [14].

Two-photon polymerization (2PP) uses a near-infrared laser to polymerize a photocurable resin [14]. This method can print structures as small as 65 nm [20] and has been used to print SU8 molds for microfluidic devices with varying heights in the z-axis [21] and open-channel microfluidic chips [22]. While this method does provide best-in-class resolution, the serial process of printing voxels results in a tradeoff between voxel size and print time. For example, the print time for a 1 cubic millimeter geometry can range from 50 h [23] to as much as 104 days [24], depending on voxel size, while microfluidic chips are often in the cubic centimeter range. Recent 2PP systems, such as the Nanoscribe QX, promise to significantly improve fabrication times with throughputs of up to 1 million voxels/second; however, their high cost limits access to the technology.

Stereolithography (SLA) is a method of 3D printing that exposes photocurable resin using a scanning or projection light source. Classical stereolithography uses a scanning UV laser source to expose one voxel at a time. Digital light projection (DLP) systems expose an entire layer at a time by projecting a 2D pattern of UV light using a collimated LED source and a liquid crystal display (LCD) panel [25]. DLP has been used to print PDMS soft lithography molds [26], open-channel microfluidic chips, which are sealed with tape post-printing [27], and entire microfluidic chips using transparent resin [27]. These devices have been used for mixing, gradient generation, droplet extraction, and chemical separation using isotachophoresis [27]. Devices exploiting 3D functionality include microfluidic valves and pumps [28], input filters, and droplet storage devices [29]. Resins have been optimized to have lower viscosity and photoinitiators of higher specificity [30] to make chips that are optically transparent and biocompatible. Generally speaking, these methods are typically able to produce minimum structures between 200 and 250 µm [27]. However, recent reports with modified SLA printers have achieved structures of 100 µm [29]. Issues with these printers include cost (high-resolution SLA costs >USD 10,000 [29]) and breakage of small structures that adhere to the floor of the liquid vat. 

Table 1 compares the fabrication methods described above in terms of cost, ability to vary the height of structures, minimum feature size, and the time to prototype a microfluidic device. We estimate the typical times to fabricate assuming moderately complex channel structures and categorize them as slow (>10 h), moderate (<8 h), and fast (<2 h). There are clear tradeoffs between resolution and fabrication time. At the high-resolution end, photolithography and 2PP can make submicron structures but require capital investments and have longer turnaround times due to mask fabrication or the time to serially print structures. Lower resolution methods such as laser cutters, Cricut, and inkjet can create structures within minutes depending on the design complexity, but minimum feature sizes are >150 µm. A key benefit of 3D printing techniques (FDM, 17, STL) is the ability to vary z-axis geometry, combined with relatively fast fabrication time. However, these methods also have low resolution (250 µm or larger). 

In this paper, we present a method to fabricate soft lithography molds for PDMS using the Phrozen Sonic Mini 8K, a commercially available high-resolution digital light projection (DLP) resin printer that costs USD 500. We describe guidelines to reliably fabricate channels as small as 44 µm wide and make smooth transitions across the z-axis. The following sections will discuss the design, print, and post-processing steps of printing molds for prototyping microfluidic devices within 1 workday (~8 h). We will also describe the limitations of the printer and propose a standard operating procedure for researchers utilizing this method for microfluidic prototyping.

## 2. Methods and Experimental Setup

**Figure 1:**
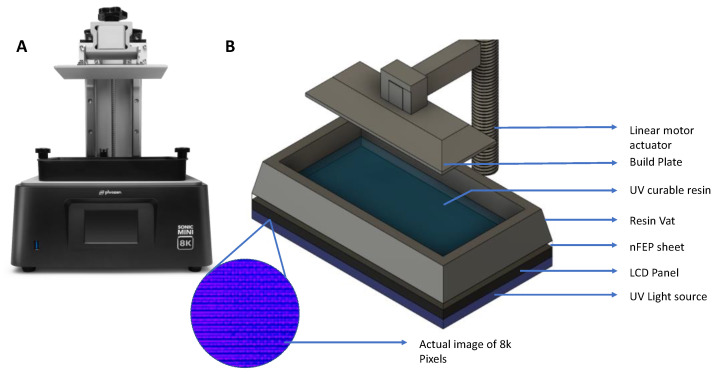
(**A**) Image of Phrozen Sonic 8K mini. (**B**) Schematic of a DLP resin 3D printer.

All molds were printed using the Phrozen Sonic Mini 8K DLP printer [25]. This printer consists of an inverted, collimated LED source emitting in the near UV spectrum (405 nm), an 8K monochromatic LCD, a vat to hold the liquid resin, and an aluminum build plate attached to a linear motor (Figure 1). The 3D model of the mold is first designed in Autodesk Fusion 360 and then sliced into thin layers (25–50 µm thick, with a 50 µm default) using the Chitubox software. The printing process consists of a series of exposure steps, one per layer. First, the inverted build plate moves to a position one layer height above the resin vat. Second, the LCD panel patterns LED illumination of the resin vat just above the panel. The exposure (405 nm, 2.8 milliwatts/cm^2^ [31]) polymerizes selected regions of the photocurable resin. The vat is coated with a 50 µm transparent fluorinated ethylene propylene (FEP) sheet, thin enough to allow light transmission yet sufficiently thick to not tear during the printing process. The anti-stiction properties of the FEP promote the release of each layer after exposure. After the exposure, the build plate first retracts upwards to separate the solidified regions from the FEP sheet and returns back down for the subsequent layer exposure. 

**Figure 2:**
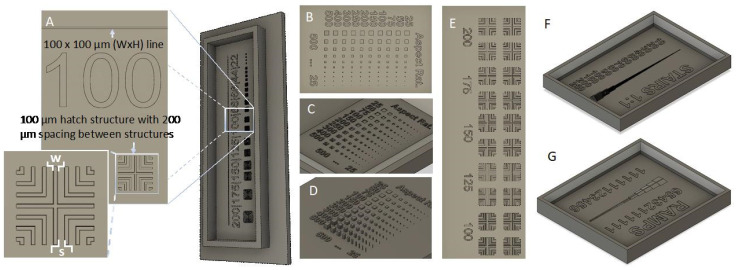
Test print design. (**A**) (Left) structure includes a long line, as well as a crosshatch structure. (right). The structure is repeated at size scales ranging from 200 to 22 µm. (**B**) Square structures of widths varying from 25 to 500 µm along the vertical axis following text and heights varying from 25 to 500 µm along the horizontal axis following text. (**C**) Indents made in the square structure. (**D**) Extrusions made in the square structure. (**E**) 100 µm crosshatch structure with spacing between structures varying from 200 to 100 µm. (**F**) Stair structures with square structures ranging from 20 to 250 µm—each having 10 stairs on each side. (**G**) Ramp structures with base-to-height ratios ranging from 6:1 to 1:6.

The resolution of the printed mold and the final part depends strongly on the resolution of the LCD panel. The Phrozen Sonic Mini 8K has a 7500 × 3240 pixels monochromatic LCD with dimensions 165 mm × 71.28 mm, resulting in an estimated pixel size of 22 µm × 22 µm. In theory, the minimum feature size may approach 22 µm; however, light scattering and lateral diffusion of photoinitiators typically increase feature size [32,33]. To determine the practical minimum printable feature, we designed L-shaped crosshatch patterns containing lines and spaces with dimensions from 200 µm to 22 µm (Figure 2A). Each structure has an aspect ratio (AR = height/width) of 1. The L-patterns have lines with width w, and space s = 2w. In the drawn dimensions, the w/s ratio is 0.5. The test structure contains multiple 90-degree turns used to evaluate the rounding of corners. To evaluate the fabrication of long microchannels, we included 2 cm long lines of widths ranging from 200 µm to 22 µm, each with AR = 1. 

Extruded mold features are typically used to create channels and chambers in a soft lithography process, while cavities are used to make pillars within the channels. We evaluated both by printing square extruded structures as well as cavities with lateral and vertical dimensions ranging from 25 µm to 500 µm (Figure 2B–D). With the extruded structures, the larger dimensions were used primarily for aspect ratio testing. To evaluate the minimum spacing between two channels, we printed multiple replicates of the aforementioned crosshatch structure with a fixed cross-section (width x height) of 100 × 100 µm^2^ and spacings ranging from 200 µm to 100 µm (Figure 2E). To evaluate vertical resolution and the ability to form variable height molds, we printed several stair structures, each with 10 fixed-size steps ranging from 10 µm to 250 µm in both lateral and vertical dimensions (Figure 2F). Finally, we printed ramp structures to test how the printer can accommodate sloping features (Figure 2G). The center spine of the ramp structures is fixed at 250 µm width and 500 µm height, while the base-to-height ratio of each ramp ranges from 6:1 (shallow ramp) to 1:6 (steep ramp). STL model files for all the test structures described in this paper can be found in the Supplementary Information. 

Unless otherwise specified, the molds were printed using the Phrozen Aqua Grey 8K resin, and the manufacturer recommended default printer settings. Notably, the manufacturer changed the recommended exposure times from 3 to 2 s during the course of the study. The default exposure time was 3 s for all experiments, except the minimum spacing study, where it was 2 s. In experiments where a print parameter (exposure time, layer height, retraction speed) was an experimental parameter, we performed the experiments in random order rather than increasing or decreasing order to reduce experimental bias. The resin was degassed prior to use with a vacuum desiccation chamber and poured carefully into the PFA-coated vat to avoid air bubbles. To test print variability across the LCD screen, each design was printed 5 times in random positions on the build plate. Imaging and dimensional measurements of the structures were performed using light microscopy and scanning electron microscopy (SEM). Microscopy measurements were performed with an Amscope adjustable zoom stereomicroscope with a 3.1 megapixel CMOS camera. SEM imaging was performed using a JOEL JSM-7600F field emission microscope. Samples were sputter coated with a nanometer-scale gold layer prior to imaging. Dimensions on recorded images were measured using ImageJ software. Dimensional scales were determined using manufacturer-provided calibration samples.

After printing the molds, we performed post-treatments to address several issues posed using 3D-printed resin molds for PDMS casting. First, the PDMS curing agent contains a platinum catalyst [34]. The cured resin can release chemicals such as polyethylene glycols, diethyl-phthalates [35], unreacted monomers [36], and phosphine-oxide photoinitiators [37], which interfere with the PDMS catalyst and inhibit curing. We followed the extensive post-print treatments outlined by Venzac [38] and developed our own recipe to reduce processing time. Second, excess resin can remain in cavities between printed structures. To clean the excess resin from the prints, we ultrasonicated the prints in isopropyl alcohol (IPA). We tested different ultrasonication times to determine whether prolonged treatment may cause structures to deform or break. After cleaning the excess resin, we exposed the print to UV light to cure any remaining resin that may be present on the surface or inner layers. We evaluated the effect of post-exposure times on how well mold structures can be faithfully replicated in PDMS. After UV exposure, the prints were baked in an oven at 60 °C for 1–8 h. Based on the findings by Venzac [38], this helps evaporate any volatile compounds that interfere with PDMS curing. Finally, we experimented with several surface treatment methods to promote easy release of PDMS after casting. 

## 3. Results and Discussion

This section discusses three groups of experiments to assess the proposed soft lithography process using 3D-printed molds. The first set (Section 3.1, Section 3.2, Section 3.3, Section 3.4, Section 3.5 and Section 3.6) evaluates the resolution and integrity of mold structures printed using the Phrozen Sonic Mini 8K. Microfluidic molds include extruded posts to form chambers, extruded lines to form chambers and channels, circular or square cavities to form posts within the channels, and long rectangular cavities to form the walls between adjacent channels. Molds may also include structures of varying heights to enable useful functions in microfluidic devices. Each of these capabilities is discussed in turn. The second set of experiments (Section 3.7, Section 3.8, Section 3.9, Section 3.10 and Section 3.11) discusses the mold post-processing techniques to ensure fidelity and faithful reproduction of structures on PDMS during the replica molding process. These include sonication and UV flood exposure to remove uncured resin that may interfere with PDMS curing, heat treatment to remove volatile compounds that inhibit curing, and nonstick surface treatments to facilitate the release of PDMS after molding. The third set describes examples of multilayer microfluidic chips that can be prototyped using the developed process. We conclude by summarizing the fabrication process and design rules. All the test structures designed and used can be downloaded from the link found in the Supplementary Information. 

### 3.1. Extruded Mold Structures

**Figure 3:**
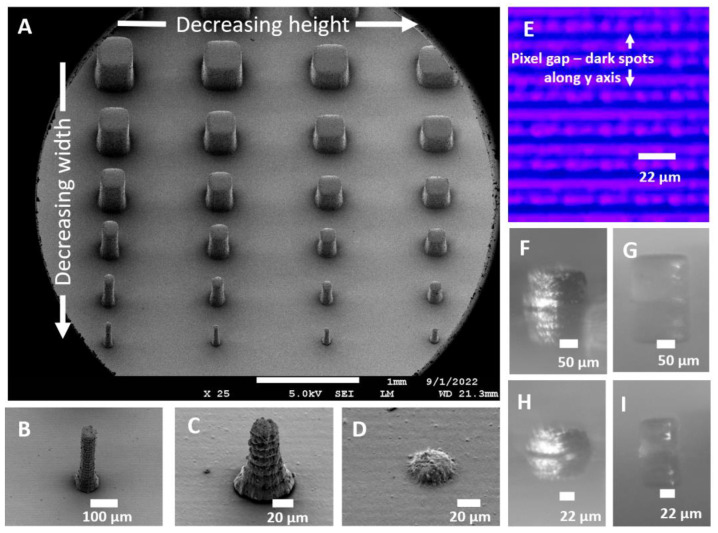
SEM image of square pillar structures. (**A**) Pillar array with varying width and height. Upper left is 300 × 300 × 500 µm^3^ and lower right is 25 × 25 × 150 µm^3^. (**B**) 75 × 75 × 500 µm^3^ pillar. (**C**) 50 × 50 × 200 µm^3^ pillar. (**D**) 50 × 50 × 25 µm^3^ pillar. (**E**) Microscopic image of Phrozen 8K Mini LCD screen. (**F**–**I**) Microscope images of cube structures. (**F**) 50 × 50 × 50 µm^3^ cube with a slice height of 25 µm. (**G**) 50 × 50 × 50 µm^3^ cube with a slice height of 50 µm. (**H**) 100 × 100 × 100 µm^3^ cube with a slice height of 25 µm. (**I**) 100 × 100 × 100 µm^3^ cube with a slice height of 50 µm.

Microfluidic molds require extruded post structures, which form cavities in PDMS after molding. Arrays of such microchambers are commonly used in high throughput parallelized assays such as single cell or digital PCR [39,40]. To determine the smallest size and best-case aspect ratio of extruded mold features, we printed an array of square posts with lateral dimensions from 500 µm to 25 µm (Figure 3A, vertical axis) and heights varying from 500 µm to 25 µm (Figure 3A, horizontal axis). To make the shallow height structures, we used a 25 µm slice height setting (vs. default of 50 µm) and a 2 s exposure (vs. a default of 3 s). 

SEM images (Figure 3) show that structures can be reproduced with some limitations. First, there is rounding of the corners in both the lateral (in-plane) dimension and the vertical (out-of-plane) dimensions near the base of the pillar. Second, layer striations on the order of 2 µm are visible on the sidewalls (Figure 3C). Third, structures smaller than 100 µm can only be printed along the x-axis because the LCD panel of the Phrozen Sonic Mini 8K printer has a pixel-to-pixel spacing of ~7 µm along the y-axis (Figure 3E). 

Finally, the smallest printable structure of 50 × 50 × 25 µm^3^ (Figure 3D) has correct lateral dimensions but a compressed z dimension. We found that the short exposure time (2 s) used with the 25 µm slice height results in partially-cured, malleable structures that may compress during the lowering of the build plate and possibly in post-print processes. Similarly, the 50 µm and 100 µm cubes are compressed to 70% of their designed height when printed with the above settings (Figure 3F,H). However, if the same structures are printed with a 50 µm slice height and 3 s exposure, they have the correct height (Figure 3G,I). The relationship between slice height and exposure time is further discussed in Section 3.4.2.

With respect to aspect ratios (AR), we found that AR > 1 can be readily obtained. For example, a 50 µm post can be printed to 200 µm height (AR = 4) (Figure 3C), and a 75 µm post can be printed to 500 µm height (AR = 6.66) (Figure 3B). However, high AR structures with narrow bases tended to delaminate when used in PDMS molding. Hence, we recommend using a base of >100 µm for structures with AR > 1.

### 3.2. Cavity Mold Structures

**Figure 4:**
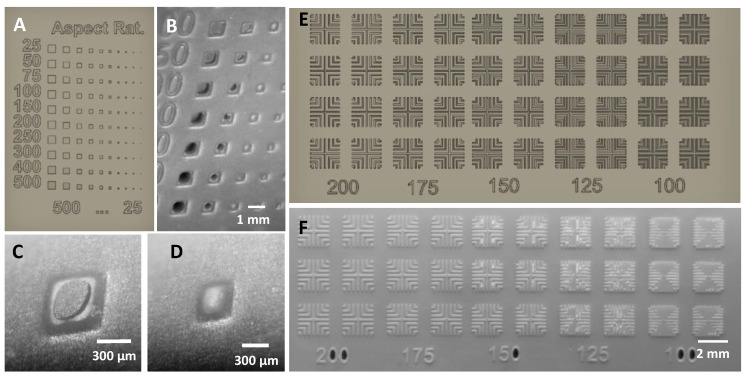
Test structures for minimum spacing. (**A**) Concept design—cavity structures in mold. (**B**) Square cavities varying from 100 µm to 500 µm after IPA wash. (**C**) 400 × 400 × 25 µm hole with base exposed. (**D**) 300 × 300 × 25 µm hole with base covered by excess resin. (**E**,**F**) Design (**E**) and photograph (**F**) of crosshatch test structures.

In addition to extruded structures, microfluidic molds typically require two types of cavity structures: cylindrical or square cavities to form support pillars within wide channels and long rectangular cavities to form the walls between adjacent channels. To determine the minimum square cavity size, we designed an array of square holes ranging from 500 to 25 µm lateral dimension and depth (shown in Figure 4A horizontal and vertical axes). Molds were imaged after printing with a 3 s exposure followed by sonication in IPA for 30 min to remove uncured resin within the cavities. 

In cavities larger than 400 µm, the uncured resin within the cavity is almost fully removed (Figure 4A), but sharp corners are rounded, likely due to the surface tension effects of partially cured resin (Figure 4B). In contrast, the 300 µm cavity (Figure 4C) remains full of uncured resin even after sonication. Although this resin may be possible to remove using high-pressure IPA washing or mechanical scrubbing, these methods may damage or deform the structures and reduce yield. We recommend that cavities be at least 400 µm wide and have rounded corners or be entirely circular. Compared to extruded structures, cavity structures must be significantly larger (50 µm vs. 400 µm).

To determine the minimum rectangular cavity size, we designed a crosshatch pattern consisting of 100 µm line structures with spacing between the structures ranging from 200 to 100 µm in decrements of 25 µm. Each pattern was printed 10 times for repeatability at 2 s exposure. The 200 µm cavity is reproduced faithfully. The 175 µm spacing shows small residues of uncured resin on corners, which reduces the actual spacing to an average of 117 +/− 1.3 µm. The 150 µm spacing exhibits bridging between the structures 8 out of 10 times, likely due to the surface tension of partially cured resin. The 125 µm and 100 µm spacing structures exhibit severe bridging between structures and are not usable. Compared to square cavities, rectangular cavities can be made significantly smaller (200 µm vs. 400 µm). Surface tension effects scale with length and are less pronounced in rectangular structures due to the longer dimension.

### 3.3. Line Structures

A third important group of mold structures includes long lines, which form channels after PDMS casting. To determine the minimum line width, we printed long lines (>1 cm length) with widths ranging from 200 to 25 µm. The channels had aspect ratios of 1. The smallest printable width was found to be 44 µm (see images in Section 3.3.2 and Section 3.3.3). Although the printer has an advertised resolution of 25 µm, the actual line width was larger (similar to Section 3.1), likely due to the effects of light scattering and diffusion of partially cured resin [32,33]. As long line structures tend to be fragile, we investigated the effects of exposure time and retraction speed on the resolution and adhesion of lines. 

#### 3.3.1. Effect of Exposure Time on Minimum Line Width and Spacing

**Figure 5:**
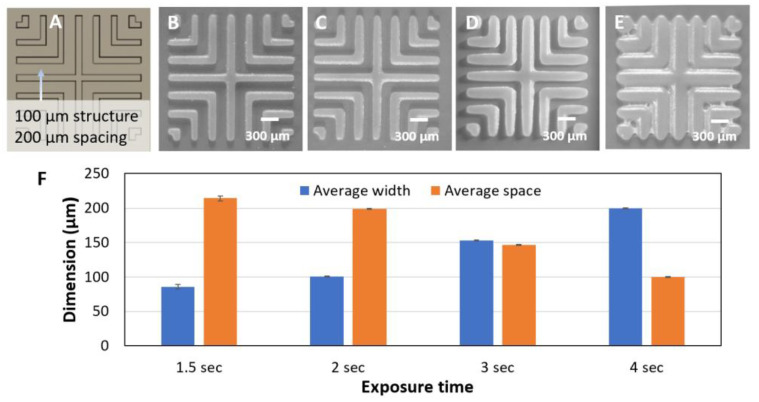
Optimization of dose. (**A**) Crosshatch test structure with 100 µm width and 200 µm spacing. (**B**) 1.5 s exposure. (**C**) 2 s exposure. (**D**) 3 s exposure. (**E**) 4 s exposure. (**F**) Comparison of exposure time vs. the average width and spacing of cross structures.

Printing high-density channels is often required in parallelized microfluidic devices and microfluidic large-scale integration [41]. Exposure time may be optimized to determine minimal channel and spacing, similar to the minimum trace and space optimization in high-density printed circuit board (PCB) manufacturing. To characterize the effect of exposure time, we printed the L-shaped hatch structures (Figure 5A) containing 100 µm traces and 200 µm spacing. We chose 200 µm spacing because smaller spaces exhibited bridging between structures at all exposure times (see Section 3.3). We compared the printed structures at several exposure times (1.5, 2, 3, and 4 s) with five replicates at each exposure time to assess process variability.

As expected, we found a linear correlation between exposure time and line width. In the 1.5 s exposure, the width is 85.9 µm +/− 6.5 µm (standard deviation, SD), about 14% smaller than the drawn dimension. The large SD may be due to the resin not being fully cured, which makes it prone to deformation during the subsequent post-processing steps. A 2 s exposure faithfully reproduces structures at 100.8 +/− 1.6µm. With a 3 s exposure, the average width is 153.3 +/− 1.5 µm, an increase of 1.5x. With a 4 s exposure, the width increases to 200 +/− 1.4 µm (~2x increase) with bridging between structures. These results are consistent with the fact that increased exposure dose expands the lateral diffraction of light and enlarges the size of structures. 

#### 3.3.2. Effect of Exposure Time on Adhesion

**Figure 6:**
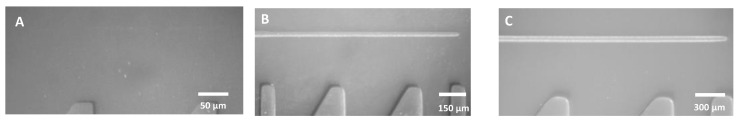
Effects of exposure time on long lines. (**A**) 44 µm line—2 s exposure. (**B**) 44 µm line—3 s exposure. (**C**) 44 µm line—5 s exposure.

Long, thin mold structures are prone to delamination, and we found that exposure time is one controllable factor. We printed a minimum dimension (44 µm) line at exposures of 2, 3, and 5 s. With a 2 s exposure (Figure 6A), the line delaminated due to the out-of-plane shear stress applied during the retraction of the build plate (see following section). With 3 and 5 s exposures, the lines remained adhered (Figure 6B,C); however, in the 5 s exposure, the measured line width increased to 84 µm due to overexposure (Figure 6C). This suggests that overexposure may improve adhesion by simply increasing the size and contact area of the structure. A 3-s exposure provides a good balance between adhesion and negligible overexposure in 44 µm lines. The results suggest that exposure optimization may be required in molds with fine lines. 

#### 3.3.3. Effect of Retraction Velocity on Delamination

**Figure 7:**
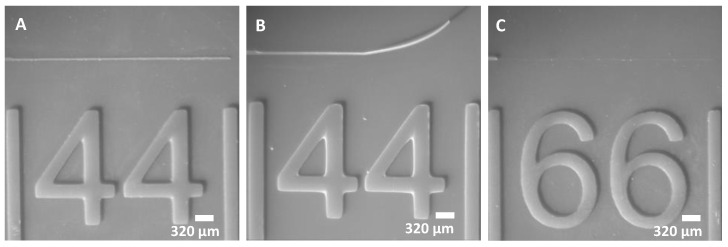
Line structure delamination. (**A**) 44 µm line remains adhered at 60 mm/min retraction velocity. (**B**) 44 µm line delaminated at 30 mm/min. (**C**) 66 µm line delaminated at 15 mm/min.

The retraction velocity is the speed at which the build plate is lifted from the nFEP sheet after each layer is exposed. During retraction, the liquid resin flows laterally into the build area from the outer regions, resulting in shear stress that can delaminate or damage fine structures. To assess this effect, we printed line structures of various widths (3 s exposure) at the manufacturer-recommended retraction speed of 60 mm/min, as well as 30 and 15 mm/min. We found that the smallest printable line was 44 µm (Figure 7A) at 60 mm/min retraction, 66 µm (Figure 7B) at half-speed, and 88 µm (Figure 7C) at quarter-speed. These results suggest that at slower retraction speeds, the longer duration of shear stress may cause delamination of long and thin channel structures.

### 3.4. Mold Structures with Varying Heights

A key benefit of 3D-printed molds is the ability to produce molds with tunable thickness, enabling microfluidic channels and structures of variable height. While multilayer molds are possible in conventional processes using SU-8 or dry film photoresists, the process requires multiple rounds of spin coating/lamination, followed by exposure and development for each layer [42]. More importantly, the alignment of layers can be challenging. Consequently, microfluidic chips are often limited to only one or a few layers. In contrast, 3D-printed molds can facilitate an arbitrarily large number of layers, and continuously varying height is also possible. Variable height enables unique capabilities in microfluidics, as will be outlined later in the paper. 

#### 3.4.1. Stair and Ramp Test Structures 

**Figure 8:**
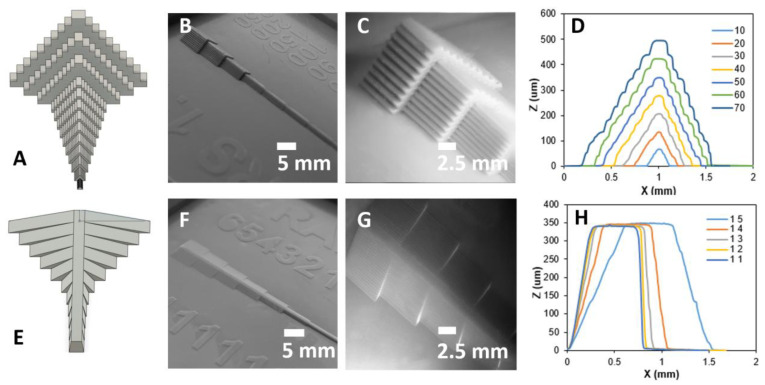
Stair and ramp test structures. (**A**) Screenshot of step design. (**B**) Photograph of step design 3D print. (**C**) Closeup of a profile taken with a stereomicroscope. (**D**) Profilometer plot of step design. (**E**) Screenshot of ramp design. (**F**) Photograph of ramp design 3D print. (**G**) Profile photo of ramp design using a boom scope (**H**) Profilometer plot of ramp design.

We designed stair and ramp test structures (Figure 8) to demonstrate the ability to fabricate discrete and continuous variations along the z-axis, with step heights as small as 25 µm. Each stair structure has 10 steps of equal height and width. To determine the resolution limits, we printed several such structures, with step dimensions ranging from 25 to 500 µm (Figure 8A,E). We then measured the surface profile with a stylus profilometer (Dektak XT) (Figure 8B–D). We repeated the same characterization for smooth, triangular-sloping structures with height-to-width ratios ranging from 1/5 to 1/1 (Figure 8F–H). 

For a 70 µm stair structure with 10 steps, the maximum height is expected to be 700 µm. However, our measurements (Figure 8D) show that the height is only 500 µm. This is likely related to using a thinner slice height (25 µm), which requires a smaller exposure to retain lateral dimensional accuracy in the xy plane (see the following sections for details). As mentioned in Section 3.1, underexposure results in partially cured resin, which may compress when the build plate lowers to the substrate. Dimensional accuracy in the z-axis can be retained using the manufacturer’s default slice height and exposure; however, this reduces the z-axis resolution to 50 µm. Another observation in both the stair and ramp structures is the rounding of sharp edges due to surface tension, as described in Section 3.2. For stairs under 40 µm, the curvature of the edge is greater than the step itself, resulting in a smoothing of the stair structure. On the other hand, the ramp structure has a more faithful reproduction of the designed print, with fewer sharp edges. The small steps in the ramp produced by slicing software are undetectable by the Dektak XT, nor are they visible in PDMS-cast chips. This suggests that discrete layers formed from slicing are smoothed during the printing and curing processes.

#### 3.4.2. Optimization of Slice Layer Thickness and Exposure

As described earlier, DLP printers expose resin layer by layer, and therefore, the slice thickness (or layer height) is an important parameter in fabricating molds with variable heights. The Phrozen Sonic Mini 8K exposure times are optimized for the default layer height of 50 µm. However, microfluidic molds typically have structures with heights ranging from 20 to 100 µm, necessitating optimization at smaller layer heights (for example, 25 µm). In thick slices, a small light dose may not fully penetrate the thickness of the layer, resulting in shallower than intended structures. Conversely, in thin slices, a large light dose may penetrate beyond the desired slice thickness, resulting in thicker structures or lateral expansion. 

**Figure 9:**
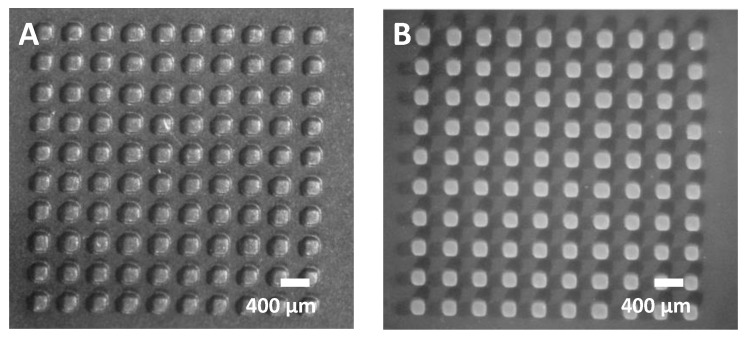
Impact of slice layer height. (**A**) 200 µm cube array—25 µm slice layer height. (**B**) 200 µm cube array—50 µm slice layer height.

We demonstrate the effect of slide layer height and exposure on an array of 200 × 200 µm^2^ square posts with 100 µm height (Figure 9). The structure was printed once at the default 50 µm slice height and once at a 25 µm slice height, with both prints using a 3 s exposure per layer. The former faithfully reproduces the 200 µm dimensions (Figure 9B), while the latter increases the lateral dimension to nearly 300 µm (Figure 9A). This can be explained by the total exposure dose: a 100 µm tall structure requires two exposures with a 50 µm slice and four exposures for a 25 µm slice. The latter receives a larger overall dose, which likely causes overexposure and expansion of structures. This experiment suggests that exposure times should be adjusted dynamically depending on the layer height of the printed structures. For example, the substrate or base of a microfluidic mold may require layer heights of ≥50 µm and correspondingly large exposure times, while the thin surface structures would require layer heights of only 10–25 µm and correspondingly smaller exposure times. While basic slicing software offers only fixed exposure times, full-featured slicers (e.g., Chitubox Pro and others) offer ‘multi-parameter’ slicing, i.e., the ability to adjust exposure time at different portions of the print. 

### 3.5. IPA Sonication to Remove Uncured Resin

**Figure 10:**
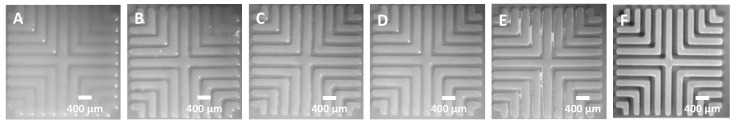
200 µm cross structures—sonication time: (**A**) 0 s, (**B**) 30 s, (**C**) 1 min, (**D**) 2 min, (**E**) 5 min, (**F**) 30 min.

The resin printing process leaves a thin layer of uncured resin on the surface of the print, which must be removed by sonication with isopropyl alcohol (IPA). Sonication time is a tradeoff between optimal cleaning of the print vs. the risk of delamination of the structures. To understand the effect, we sonicated a 3 s exposure print in IPA for 0 s, 30 s, 1 min, 2 min, 5 min, and 30 min in a Branson ultrasonic cleaner. Treatments below 2 min do not fully remove resin from the mold (Figure 10A–C), while at about 2 min, molds show minimal residue (Figure 10D). A 5 min treatment appears to remove all residue (Figure 10E); however, structures smaller than 50 µm delaminate. Similarly, 30 min treatments remove all residue (Figure 10F) but delaminate structures smaller than 66 µm, similar to results discussed earlier in Figure 6A and Figure 7B,C. For molds with structures >100 µm, a 30 min treatment is recommended to provide the cleanest possible mold. 

Although our experiments used IPA, we also evaluated other solvents. More aggressive solvents are effective in removing uncured resin but may damage or delaminate fine structures. The resins, in order of least to most aggressive, include IPA, ethanol, acetone, acetonitrile, and chloroform. When using chloroform, it is limited to contact to <30 s and immediately blot with a microfiber cloth to rapidly absorb the solvent and resin.

### 3.6. UV Curing to Eliminate Photoinitiators

**Figure 11:**
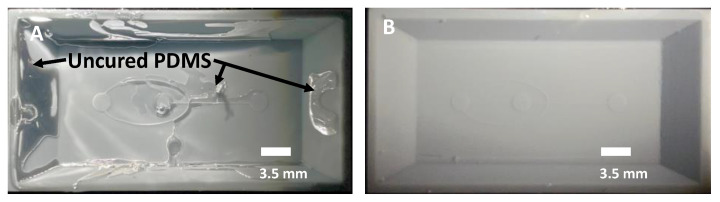
PDMS cast on-chip with post-print UV exposure time of (**A**) 15 min and (**B**) 30 min.

Molds must be treated with a UV flood exposure after IPA sonication to fully cure any photoinitiators remaining on the mold after printing. This is a critical step [28] since phosphine oxide-based photoinitiators interfere with the Pt- based PDMS curing catalyst and inhibit curing of the PDMS during soft lithography. To understand the effect of flood exposure on PDMS curing, we cast PDMS from a simple cross-channel mold structure with a 3 s print exposure. We exposed the molds to UV light for 0, 5, 10, 15, and 30 min and then heated each mold in an oven at 60 °C for 1 h. With a 15-min exposure (Figure 11A), patches of uncured PDMS cannot be removed from the sidewalls of the mold, resulting in poor replication. Increasing the flood exposure to 30 min produces fully cured PDMS (Figure 11B). 

### 3.7. Heat Treatment to Promote PDMS Curing

**Figure 12:**
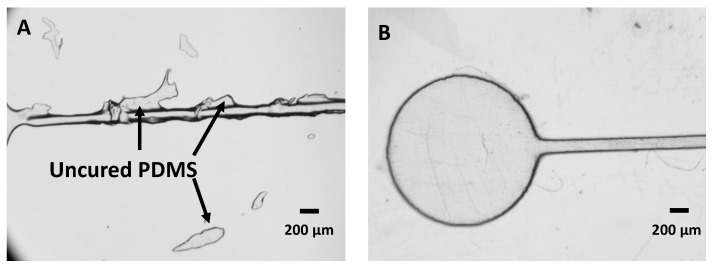
PDMS cast on a mold with post-print heating for (**A**) 0 min and (**B**) 60 min.

Soft lithography molds must be heated for a minimum time period to fully remove volatile impurities that may interfere with PDMS polymerization [38]. We exposed two molds to UV for 30 min and then baked one mold at 60 °C for 1 h while the other was unheated. We then coated these molds with silane (Section 3.8) and casted PDMS. With no heat treatment, patches of uncured PDMS remain adhered to the mold, resulting in poor reproduction of the mold structures (Figure 12A). In contrast, the mold that was heated for 1 h at 60 °C produced a faithful reproduction of the mold with accurate dimensions of a 100 µm channel and a 1.5 mm outlet (Figure 12B).

### 3.8. Anti-Stiction Coating to Facilitate PDMS Release

**Figure 13:**
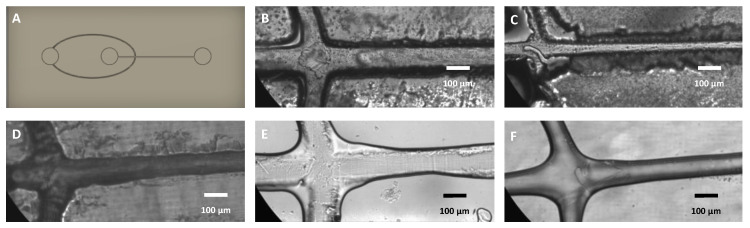
Effects of mold coating on droplet generator. (**A**) Drop generator design. (**B**) PDMS cast on a non-coated chip. (**C**) PDMS cast on mold sprayed with nonstick silicone. (**D**) PDMS cast on mold dipped in FC40. (**E**) PDMS cast on mold dipped in silicone oil. (**F**) PDMS cast on mold treated with silane.

Molds used for PDMS casting are typically treated with a nonstick coating to promote the release of the cured PDMS. Among the several options we tested, we found that vapor deposition of trichloro (1H,1H,2H,2H—perfluorooctyl) silane provided the best release. For this test, we used a simple droplet generator design consisting of two 100 µm × 100 µm channels (w × h) intersecting at a cross junction and leading into another 100 µm × 100 µm channel (w × h) and then to the outlet. The mold has two inlets (oil, left, and water, middle) and an outlet (right), all with a 1.5 mm diameter. When no coating was applied, the cured PDMS partially adhered to the mold and was damaged when removed, leading to poor optical quality and uneven surface in the microfluidic channel (Figure 13B). We attempted several coatings to release the PDMS from the mold: (1) a spray-on silicone release layer (Dow Corning 316) (Figure 13C); (2) soaking the mold in fluorocarbon oil (FC-40, 3M) for 30 min (Figure 13D); (3) soaking the mold in Dow Corning silicone oil for 30 min (Figure 13E), and finally (4) vapor deposition of Tricloro (1H,1H,2H,2H—perfluorooctyl) silane for 30 min (Figure 13F). In methods 2–4, the soak was followed by sonication in IPA for 30 min. The first three methods inhibited PDMS curing and, in the case of silicone oil, caused swelling of the channel. The vapor-phase silane treatment provided easy release and channels with smooth sidewalls and favorable optical quality.

### 3.9. Sidewalls to Prevent Mold Warpage

Microfluidic molds made from resin printers have a tendency to warp due to non-uniform surface stresses that evolve during the print and post-print curing processes. For example, warping can occur due to inhomogeneous curing of photoinitiators during UV flood exposure. For example, if the curing light is placed on the top side of the mold, that side will be more exposed than the underside since the mold is not transparent. The exposed plane experiences a higher cure rate, which generates tensile stress that deflects the print toward that side. Washing in IPA may also induce temperature shifts which change the volume of the printed structure [43]. 

**Figure 14:**
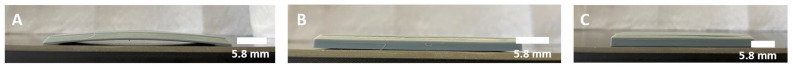
Effect of sidewalls on warping, showing mold with (**A**) no side wall—extreme warping, (**B**) 1 mm sidewall—mild warping, (**C**) 2mm side wall—no warping.

We found that sidewalls of sufficient thickness reduce warping. Figure 14 shows microfluidic molds with no side wall (Figure 14A), 1mm side wall (Figure 14B), and 2 mm sidewall (Figure 14C) for a design that was 70 × 25 mm (L × W). In this design, the base layer is 3 mm thick. With no sidewall, the chip experiences significant warping. In this mold, the side of the chip (lower side in picture) exposed to UV exhibits the characteristic contraction. With a 1 mm side wall, we see minimal to moderate warping. In this image, the upper side was exposed to UV, and the print bends upward. A 2 mm sidewall prevents warping entirely. The thickness of the sidewall provides rigidity to maintain shape despite the surface stresses. 

The minimum required thickness of the sidewall depends on the thickness of the base layer. Thinner base layers (<3 mm) are more likely to warp, and the sidewall height must be increased accordingly. Base layers that are 4 mm thick behave similarly to 3 mm bases. We did not print base layers >4 mm as adding more layer thickness increases print times and resin consumption.

### 3.10. Applications: Microfluidic Devices Fabricated from 3D Printed Molds

**Figure 15:**
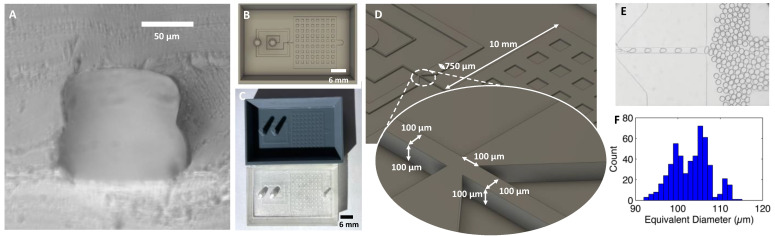
(**A**) Cross section of 100 × 100 µm PDMS channel cast using the 3D printed mold. (**B**) Screenshot of chip design. (**C**) 3D printed mold and PDMS replica. (**D**) Droplets generated by the chip. (**E**) Histogram of the droplet diameter. (**F**) Diameter histogram of the droplets generated.

To demonstrate the ability to mold microfluidic chips from this process, we fabricated a variety of designs, from droplet generators to cell sorters (STL model files for all designs are available in Supplementary Information). The entire fabrication process required 8 h, starting from mold printing to PDMS curing and bonding. In comparison, the time for a typical SU8 process is about 4–5 days. Furthermore, the 3D-printed molds can include inlets and outlet reservoirs, eliminating the manual and imprecise process of punching inlets and outlets. The sidewalls serve as an integrated tray which reduces the PDMS consumption and eliminates the manual step of cutting PDMS molds with a knife. Compared to FDM printed chips, the chips have smoother sidewalls (Figure 15A) and better optical and biocompatibility of PDMS [16]. 

Our first example is a simple single-layer droplet generator (Figure 15) consisting of a cross junction with a 100 × 100 µm^2^ cross-section. We recorded videos of drop generation and used our GPU-accelerated droplet analysis software to obtain a distribution of the drop size [44]. The cross-section of the PDMS chip (Figure 15A) shows smooth sidewalls and two 50 µm print layers. The pressure-driven drop generator produces monodisperse drops with a mean diameter of 105 µm and a standard deviation of 8 µm (Figure 15B–E).

**Figure 16:**
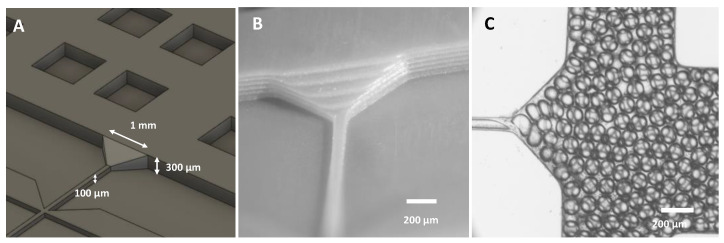
Ramp emulsifier. (**A**) Screenshot of CAD design. (**B**) Photograph of 3D printed mold. (**C**) Image of ramp emulsifier generating monodisperse droplets.

In the second example, we exploited the 3D variable height capabilities of the microfluidic molds by printing a ramp emulsifier (Figure 16A,B). The mold is similar to the step emulsifier design described by Li [45], in which a 100 × 100 µm^2^ channel drains into a 300 µm tall reservoir. Step emulsifiers are a more robust way of generating droplets but require more effort in mold fabrication as it is a multilayer design. Step emulsifiers [46,47] typically have a lateral expansion followed by a vertical expansion. Here, we demonstrate increasing both simultaneously (Figure 16C), which enables natural isotropic expansion of the droplet and allows tunability of the drop generation system.

**Figure 17:**
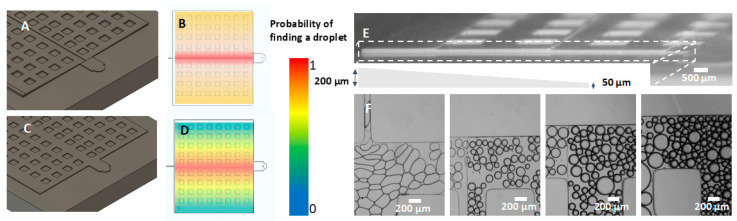
Sloped height reservoir. (**A**) CAD design. (**B**) Probability estimation of finding droplets in sloped height reservoir. (**C**) Screenshot of uniform height reservoir. (**D**) Probability estimation of finding droplets in the uniform reservoir. (**E**) Photograph and graphic representation of a cross-section of the sloped height reservoir. (**F**) Droplets in various sections of the sloped height reservoir.

To further explore the sloped channel capabilities of the 3D-printed molds, we printed a mold with a sloped height reservoir (Figure 17). In reservoirs of uniform height, droplets tend to fill the reservoir along the path of least hydraulic resistance—a straight path to the outlet—and then slowly fill the rest of the reservoir. As a result, filling an entire reservoir can become time-consuming, and during this time, many droplets are lost through the outlet. To address this issue, we designed a reservoir where the middle section has 50 µm height, and the height increases laterally to 200 µm in steps of 50 µm. We found that this reservoir design fills 3–4x faster, resulting in fewer droplets escaping via the outlet. As droplets are injected into the middle of the channel, the short channel height results in them having a flattened ‘pancake’ shape with high surface energy. Due to the lateral increase in channel height, the droplets naturally flow outwards so they can expand to a spherical shape with lower surface energy. This migration results in a more uniform distribution of droplets in the reservoir, faster filling, and reduced sample loss. 

**Figure 18:**
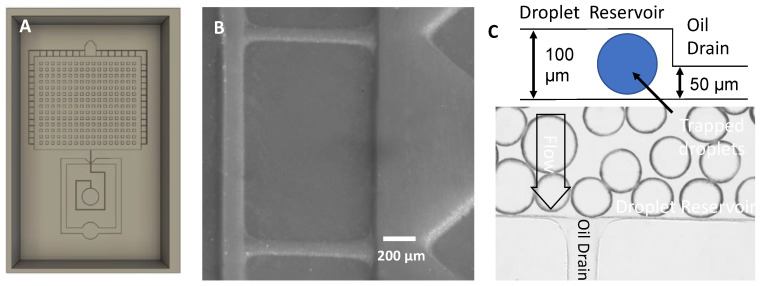
Droplet reservoir with gutters. (**A**) Screenshot of chip design. (**B**) Photograph of 3D-printed mold—50 µm tall drain section. (**C**) Image of a 50 µm tall drainage system in a 100 µm tall droplet chamber—inset shows a conceptual diagram of the filter drain design.

In droplet microfluidics, it is common to generate drops and then image them in a reservoir where the drops are held stationary in single-layer, two-dimensional arrays. To accommodate this, we printed a 100 µm-tall droplet reservoir shunted by multiple drain/gutter channels with 50 µm height (Figure 18A–C). If moderate suction is applied to the outlet, the droplets are trapped in the tall reservoir while the oil can flow through the shallow drain channel. This design enables the oil flow to pull the droplets into a packed array and illustrates the value of a simple z-axis geometry feature that provides a useful retention function in multiphase microfluidics.

**Figure 19:**
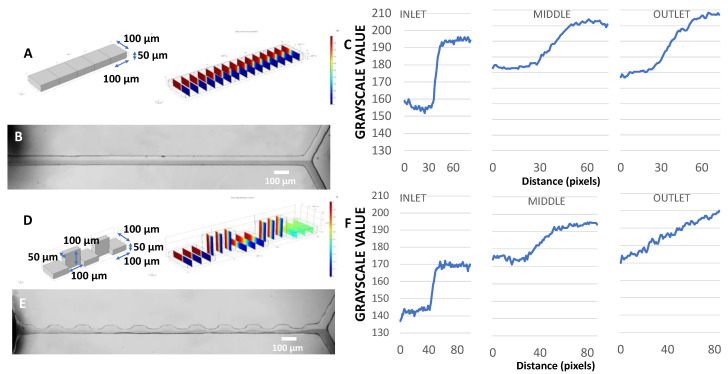
Variable aspect ratio mixer. (**A**) COMSOL simulation of conventional mixer with uniform cross-section. (**B**) Mixer fabricated using the prototyping process. The mixer combines two streams, one containing food dye. (**C**) Grayscale values across the channel at various locations along its length. (**D**–**F**) Similar plots are shown for mixers with varying aspect ratios.

Finally, we demonstrate an application beyond droplets: an efficient mixer that exploits a varying channel aspect ratio made possible by 3D-printed molds. Microfluidic mixers mix two or more streams entering a junction. In the laminar flow regime, molecular mixing happens only by diffusion, which is dependent on the area of interaction between the two streams. As shown in Figure 19A–D, increasing the aspect ratio of a channel not only decreases the average distance between the two laminar streams but also increases the area of interaction, thereby enhancing diffusive mixing. This is similar in principle to flow-focusing-based mixers [48] while addressing a key limitation. In flow-focusing devices, the narrowed ‘flow focusing’ region has a smaller distance between the streams but also has a larger velocity, which requires a longer channel to maintain sufficient interaction time between the streams. The advantage of the variable aspect ratio design shown is that the velocity remains the same because the total cross-sectional area does not change. Grayscale analysis of mixing (Figure 19C) shows that a basic channel mixer (100 µm × 100 µm × 10 mm) still has plateaued grayscale values at the end of the segment, indicating incomplete mixing of the two streams. The varying aspect ratio design consists of alternating segments with cross sections of 50 µm × 100 µm × 1 mm and 100 µm × 50 µm × 1 mm (w × h × l), respectively. This design (Figure 19F) shows a small grayscale difference between the two streams at the outlet, suggesting more efficient mixing. These results match COMSOL simulations comparing mixing in similar channel geometries and conditions. The CFD simulations were performed in COMSOL using the laminar flow and chemical diffusion modules. Identical diffusion coefficients were used in both modules. The simulations and experiments essentially show that a smaller average characteristic diffusion length in the variable aspect ratio improves mixing performance.

### 3.11. Solvent Recycling

**Figure 20:**
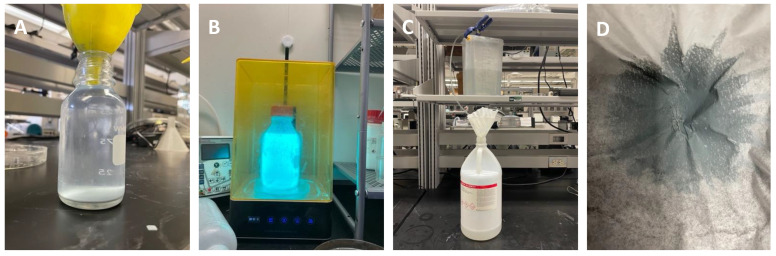
IPA Recycling. (**A**) IPA contaminated with 3D printing resin (**B**) exposing contaminated IPA to UV. (**C**) Siphoning setup used to retrieve clean IPA. (**D**) The residue left on the coffee filter after filtering IPA treated with alum.

The process described in this paper, and 3D printing in general, typically consumes large volumes of isopropyl alcohol (IPA) due to multiple rounds of solvent wash steps. As the solvents become contaminated over time, resin particulates can deposit themselves on the print during washing steps, resulting in artifacts and debris in the PDMS replicas. Frequent replacement of large volumes of solvent (often several liters or more) can increase the cost and environmental impact of the proposed process. Accordingly, we suggest strategies to recycle IPA to lower costs and environmental waste (Figure 20). 

The first step is to precipitate or coagulate the resin using one of two methods. The first is UV curing. If the contaminated IPA has a high concentration of solubilized resin, it can be exposed to UV light, which cures and precipitates the resin (Figure 20B). The flood exposure can be performed in a typical wash and cure station used during the printing process. The alternative method is coagulation. Adding aluminum sulfate (alum) has been shown to coagulate the resin particles [49] by a process similar to that used in wastewater treatment [50]. Since alum is insoluble in IPA, it must first be added to water before adding to the solvent. This method is quite effective but requires additional materials and contaminates the solvent with water. 

Once the resin has been precipitated or coagulated, the particulates can be removed using a number of methods. A simple approach is to filter the solvent through coffee filters, which have a typical pore size of 20 µm. A drawback is that very small resin particulates may not be removed. A second approach is sedimentation. The solvent can be left to settle overnight, allowing the large particles to sediment to the bottom of the container. The clean IPA is siphoned or decanted from the top and then filtered as before to remove additional particulates. Although this method is quite simple, a drawback is that small particulates with sedimentation times of days or weeks may remain in the solvent. The third approach is distillation, which involves heating the solvent above its boiling point and using a distillation column to capture the condensed solvent. This method requires a distillation apparatus and hotplate but is highly effective at purifying the IPA from resin and water contaminants. Regardless of the method used, the resin waste from this process should be disposed of per chemical waste guidelines.

## 4. Summary and Conclusions

In this paper, we described a 1-day process for making sub-100 µm microfluidic channels using soft lithography molds fabricated from a sub-USD 600 3D printer. We anticipate that this method will provide a balance of resolution, multilayer capability, cost, and turnaround time that will be attractive to many labs. 

Table 2 describes the various steps in the fabrication process. It also gives the minimum and maximum time required for each step in the fabrication process. In our experiments, the post-printing steps required only the minimum time mentioned in the table. Larger molds may require longer process times. 

Summarizing our experiments and observations above, we recommend the following guidelines for mold printing and chip fabrication.

- The recommended channel width for highly reproducible printing is ~100 µm. Printing at 50 µm is possible, but there may be higher batch-to-batch variability.
- The minimum gap between square or circular cavity structures is 400 µm. To avoid the rounding of cavity corners, circular cavities are preferred.
- The minimum distance between adjacent microchannels can be as small as 200 µm.
- The minimum Z height for reproducible printing is 25 µm, using a 25 µm slice layer height. For taller structures, we recommend using the default 50 µm slices and exposure time to avoid extra exposures due to smaller slices.
- The optimal exposure time for the Phrozen Aqua Grey 8K resin is 3 s for a 50 µm slice layer height. Over and underexposure may result in larger structures (albeit for different reasons).
- The mold base under the channels should be at least 3 mm thick to avoid warping or breakage of the base layer.
- The side walls should be at least 2 mm tall to avoid the warping of the mold. Larger chips (for example, 35 × 85 mm) may require 5 mm sidewalls.
- Post printing, the chips should be pre-rinsed with IPA, using a squeeze bottle to reduce contamination of the IPA bath. They can then be submerged in an IPA bath and sonicated for 15 min at room temperature. Too little time or ultrasonic power may result in uncured resin left in the mold, while too much time may delaminate fine structures below 50 µm.
- After 15 min, the IPA bath should be replaced with fresh IPA, followed by another 15 min of sonication. If any visible residue is observed, we suggest another round of sonication with fresh IPA for 15 min.
- The chips must be exposed to UV light for at least 30 min to cure any excess photo initiators that may interfere with PDMS curing.
- After exposure, the chips must be baked in an oven for 1 h at 60 °C to remove any additional volatile impurities that may interfere with PDMS curing.
- If uncured PDMS is observed after the molding process, consider increasing the duration of the UV exposure and heat treatment.
- Vapor deposition of trichloro (1H,1H,2H,2H—perfluorooctyl) silane should be performed on the chips for 30 min to provide an anti-stiction coating that facilitates the removal of PDMS from the mold. After coating, the chips must be placed in an IPA bath and sonicated for 30 min to remove any excess silane before PDMS casting.
- After the mold has been fabricated, soft lithography can be performed using typical procedures. A detailed standard operating procedure (SOP) is provided in the Appendix A.

## Figures and Tables

**Table 1:** Comparison of common methods for fabricating microfluidic chips.

	Device < $USD 1000	Z Axis Variation	Minimum Feature	Mold/Chip	Time to Fabricate
Photolithography [7]	No	Hard	50 nm	Mold	Slow
FDM [16]	Yes	Easy	40 µm	Both	Moderate
2PP [21]	No	Easy	110 nm	Both	Slow
i3DP [17]	No	Easy	250 µm	Mold	Moderate
DLP [28]	No	Easy	100 µm	Mold	Moderate
Laser Cutter [12]	No	Hard	250 µm	Chip	Fast
Cricut [11]	Yes	Hard	200 µm	Chip	Fast
Inkjet printing [13]	Yes	Hard	150 µm	Chip	Fast
8K LCD resin printer	Yes	Easy	44 µm	Mold	Moderate

**Table 2:** Time required for each fabrication process step.

Process Step	Minimum Time Required (Minutes)	Maximum Time Required (Minutes)
Fusion 360 design	10	60+
3D printing	25	60+
IPA wash 1	30	60
UV exposure	30	60
Heat treatment	60	480
Silane vapor deposition	20	30
IPA wash 2	30	30
PDMS casting	60	90

## Supplementary Materials

The following supporting information can be downloaded at https://www.mdpi.com/article/10.3390/mi14081519/s1: CAD files (.stl) for the test structures and molds presented in this paper.

## Appendix A. Standard Operating Procedure (SOP)

List of equipment and materials

- Phrozen Sonic Mini 8K 3D printer
- Phrozen 8K aqua grey resin
- N-FEP sheet compatible with Phrozen Sonic Mini 8K
- Computer with Chitubox slicing software
- Deiner electronic Zepto vacuum plasma chamber
- Vacuum desiccator
- Branson ultrasonic cleaner
- Elegoo wash and cure station, or similar system
- Temperature controlled oven
- Sylgard 184 PDMS elastomer kit
- IPA
- Trichloro(1H, 1H,2H, 2H-perfluorooctyl)silane
- 500 mL glass beaker
- Glass dish (Corning petri dish)
- 1.5 mm biopsy punch
- 60 mL syringe

**Preparing resin for printing**

- Shake the bottle of Phrozen 8K vigorously to homogenize the resin.
- Pour the resin into A 500 mL beaker and place it in the desiccator for 1–2 h to remove air bubbles. Check to make sure no air bubbles are formed on top.
- Pour the resin carefully into a corner of the vat, taking care not to incorporate any air bubbles into the resin. Air in resin would cause air bubbles and will result in improper prints.

**CAD design file**

The CAD design for the chip can be performed in any software that produces STL format. We used Autodesk Fusion 360. Chitubox is a slicing software compatible with the Phrozen Sonic Mini 8K printer. Other compatible slicing software may be used.

- Export the CAD design to STL format.
- Open Chitubox and click the settings button.
- Add new printer—if Phrozen 8K sonic mini is not found, use the following settings:
  ○ X/Y resolution 7500 × 3240 px;
  ○ Lock Ratio: on
  ○ Size X/Y/Z: 165 mm/71.28 mm/180 mm
  ○ Build Area Offset: off
  ○ Mirror: LCD mirror
- If using a configuration file downloaded from the internet, click the import profile button from the top right. Select the config file for the resin being used. Select resin and close the window.
- Drag the STL file into the Chitubox window. This action pastes the design on the print area. Repeat this step to arrange and print multiple objects at once.
- Click slice and save the .ctb file. This file is copied to a USB drive and used for printing.

**Calibrating settings**

This section may be performed if you wish to optimize exposure time.

- Calibration settings may be changed in the Chitubox print Table.
- The calibration settings for several resins may be found at https://phrozen3d.com/pages/resin-mini-8K (accessed on 1 June 2023).
- If the calibration file for a given resin is not found, use the sample calibration file provided in the Supplementary Information ‘*3D printing test structures_4mm base v1.stl*’.
- While printing the calibration file, check for manufacturer settings and print the design at the nominal exposure time. Reprint the design at +/− 1 s exposure.
- Observe the printed structures under a microscope to determine the optimal exposure time.
  ○ If the structures and the gaps are the same width, use that exposure time.
  ○ If the structure is larger than the gap, this is overexposed. Use a shorter exposure time.
  ○ If the structure is smaller than the gap, it is underexposed. Use a longer exposure time.

**Changing the nFEP sheet**

The nFEP sheet placed on the LCD screen protects it and facilitates the release of resin layers after exposure. The sheet must be replaced when blemishes or defects are observed.

- Remove the 4 corner locating screws.
- Remove the tensioning screws of the screen holder in an alternating manner, one half turn at a time. This step is to ensure even tension is released and thus minimizes the potential for warping.
- Once the screws are removed, flip the cover over and remove the screws that hold the sheet to the frame.
- Place all screws in the IPA to clean any residue.
- Place the new nFEP sheet (cut to size) on the frame. Once sandwiched, use a hot soldering iron or sharp tip to puncture screw holes. The frame will act as a guide for the screw holes.
- Tighten all the screws in the same order as above. Note that it is normal for the nFEP sheet to be loose in the frame. Once it is tightened to the vat walls, tension is applied by the wall design.

**Mold printing**

- Connect the USB with the .ctb file to the 3D printer. The .ctb file must be in the root directory.
- Press print on the touch screen to begin.

**Mold post-processing**

- Remove the build plate from the printer. Hang it over the vat at about a 90-degree angle and drain excess resin back into the vat for 30 min.
- Detach the print from the build plate. Use a plastic spatula to avoid scratching the build plate.
- Place the prints in a glass dish with channels facing down so that any sediment falls from the chip. Cover the print with IPA.
- Place the dish in the bottom of the ultrasonic bath and run for 15 min.
- Replace with clean IPA, and run the ultrasonic cleaner for another 15 min.
- Remove the print from the IPA and air dry.
- Place print on top of an inverted glass beaker inside the Elegoo wash and cure station or similar system. The beaker will expose the bottom side of the print to UV. Perform UV exposure for 30 min to cure any remaining uncured resin.
- Heat the mold in the oven at 60 °C for 1 h to remove any volatile components that may inhibit PDMS curing.
- Place the mold in a vacuum desiccator. In a small beaker, add 500–750 µL (depending on the number/size of molds) of trichloro(1H, 1H,2H, 2H-perfluorooctyl)silane. Turn on the vacuum for 20–30 min.
- Perform an ultrasonic wash for 30 min with the mold upside down to remove any excess silane.

**PDMS molding**

These steps can be started while the mold is being exposed to silane.

- Remove the plunger from a 60 mL syringe and close the mouth with a stopper.
- Pour PDMS and curing agent in a 10:1 ratio into the syringe.
- Mix thoroughly for 10 min using the helical mixer attachment.
- Place the syringe in a desiccator under vacuum for 1–2 h to remove all bubbles.
- Carefully put the plunger back while removing the stopper from the mouth.
- Once the molds are ready, pour PDMS carefully into the molds without introducing air bubbles. If air bubbles are introduced, desiccate for 20–30 min in a vacuum.
- Cure PDMS for 60 min at 65 °C in a laboratory oven. Cover with glass to avoid bubbling.

**PDMS removal and chip bonding**

- Remove the PDMS from the mold carefully by running a razor blade through the edges and gently lifting. Work slowly to avoid ripping the PDMS, and use a sharp razor blade to avoid fraying the edges. Excessive fraying may indicate a poor silanization process.
- Place a piece of tape on the top and bottom of the PDMS to prevent dust buildup before bonding.
- If the mold does not already include reservoirs or holes for fluidic connections, use a biopsy punch to bore the appropriately-sized hole for tubing. For example, use a 1.5 mm biopsy punch for 1.6 outer diameter (OD) tubing.
- Turn on the plasma treater, ensuring that the exhaust of the plasma system is vented outdoors or in a fume hood. Vent the plasma chamber to atmospheric pressure. Remove the chamber lid and place the PDMS with the channel side to be bonded facing upward. In the chamber, add a glass slide or flat piece of PDMS to which the molded PDMS channel will be bonded. It is helpful to place a small piece of tape to mark which side is exposed to the plasma. The side facing up will have optimal plasma treatment.
- Replace the plasma chamber lid and activate the vacuum to pump down the chamber. Hold lid in place until vacuum is established (about 10 s). After 10 min, open the oxygen flow valve for 30 s. Set flow rate at 1.0 nl/h.
- Turn on the plasma RF generator for 10–15 s. Ideally, the process gas in the plasma chamber will have deep blue/purple color, indicating the formation of ozone. A mildly purple glow indicates the presence of some N_2_ but will still perform sufficient surface functionalization for bonding. However, a pale blue color suggests that O_3_ is not being formed, and the chip may not bond. Excessively long treatments may damage the PDMS.
- Once the exposure is complete, turn off the generator, turn off the vacuum, and vent the chamber. Place the PDMS on the glass slide and gently apply pressure without collapsing channels.
- To strengthen the bond, heat the chip in an oven at 35 °C for 10 min.
